# Muscular and glenohumeral changes in the shoulder after brachial plexus birth palsy: an MRI study in a rat model

**DOI:** 10.1186/1749-7221-7-9

**Published:** 2012-12-06

**Authors:** Francisco Soldado, David Benito-Castillo, Cesar G Fontecha, Ignasi Barber, Mario Marotta, Sleiman Haddad, Mariano E Menendez, Vasco V Mascarenhas, Scott H Kozin

**Affiliations:** 1 Vall Hebron Research Institute (VHIR), Universitat Autònoma de Barcelona, Barcelona, Spain; 2 Orthopedic surgery. Fundació Hospital de l'Esperit Sant, Barcelona, Spain; 3 Paediatric Radiology Department, Barcelona, Spain; 4 UIME, Centro de Imagiologia, ES Saude Lisboa, Hospital da Luz, Lisboa, Portugal; 5 Shriners Hospital for Children. Department of Orthopaedic Surgery, Temple University & Hand Surgeon, Philadelphia, PA, USA

**Keywords:** Shoulder anomalies following brachial plexus birth palsy, Shoulder internal rotation contracture, Glenohumeral dysplasia, Erb’s palsy

## Abstract

**Background:**

Shoulder abnormalities are the major cause of morbidity in upper brachial plexus birth palsy (BPBP). We developed a rat model of upper trunk BPBP and compared our findings to previously reported animal models and to clinical findings in humans.

**Methods:**

Forty-three 5-day-old newborn rats underwent selective upper trunk neurectomy of the right brachial plexus and were studied 3 to 20 weeks after surgery. The passive shoulder external rotation was measured and the shoulder joint was assessed bilaterally by a 7.2T MRI bilaterally.

**Results:**

We found a marked decrease in passive shoulder external rotation, associated with a severe subscapularis muscle atrophy and contracture. None however developed the typical pattern of glenohumeral dysplasia.

**Conclusions:**

In contradiction with previous reports, our study shows that the rat model is not adequate for preclinical studies of shoulder dysplasia. However, it might serve as a useful model for studies analyzing shoulder contracture occurring after upper BPBP.

## Introduction

Shoulder abnormalities are the most common long-term complication and the major cause of morbidity in upper trunk brachial plexus birth palsy (BPBP) [1]. Shoulder functional impairment is due to a progressive development of muscle abnormalities, with shoulder internal rotation contracture leading to major joint deformities (i.e. glenohumeral dysplasia) [2-4]. Shoulder joint deformity has been extensively studied while the pathogenesis of muscular changes remains barely understood [5-7]. An animal model of shoulder disorders after BPBP would permit the assessment of these deformities, help define the muscle changes and would also provide a base for treatment research. Previous murine models only focused on joint changes after upper brachial plexus trunk neurectomy and came up with equivocal conclusions [8-10]. The purpose of this study is to use a similar rat model, combined with MRI evaluation, to better delineate muscle and joint shoulder changes after BPBP. We would also discuss its usefulness as a preclinical model.

## Methods

This study was carried out following the National Institutes of Health Guidelines for the use of laboratory animals and with the approval of the local Ethics Committee for experimental animal use. Forty three rat newborns from four pregnant Sprague-Dowley OFA rats were used in this study. Four rat pups died during or after surgery, and were removed from the study.

### Rat newborn surgery

5-day-old rat pups underwent right brachial plexus surgery under general anaesthesia with isofluorane. A surgical microscope was used for dissection. A transverse incision below the clavicle with splitting of pectoralis major and pectoralis minor muscles was done to expose the brachial plexus. By locating the suprascapular nerve, we reached the upper trunk of the brachial plexus. A segmental excision of the upper trunk using microscissors was then performed to simulate upper trunk neurtometic injury.

### Clinical evaluation

Glenohumeral passive external rotation range of motion was evaluated immediately after sacrifice and right before MRI evaluation. The neutral position of the shoulder was defined as the shoulder in 0° abduction and the elbow in 90° of flexion with the front limbs up ventrally, perpendicular to the examination table. After scapular stabilization with the thumb, external glenohumeral rotation was measured using a goniometer. The left side was also measured and used as a control.

### MRI evaluation

All animals were sacrificed using sodium pentobarbital injected intraperitoneally after sedation. The animal was then placed into a 7.2T MRI Biospect system (Bruker, Germany) in supine position with the front limbs onto its abdomen. Both shoulders were independently evaluated using RARE 1mm axial TR 4000 T 30 sequence in axial and sagital oblique planes parallel to the scapula. Each shoulder was analyzed independently. MRI was obtained at different times after surgery: 10 rats after 3 weeks, 10 rats after 4 weeks, 7 at 8 weeks, 6 at 15 weeks and 6 at 20 weeks. MRI analysis included mesurement of the glenoid version, PHHA (percentage of the humeral head anterior to the middle of the glenoid fossa), humeral head section area and infraspinatus and subscapular muscle thickness. As a measurement tool, we used Osirix (Apple). All mesurements were done by the same examiner.

### Statistical analysis

In order to improve statistical power, rats were grouped into two groups. The early group (n=20) included the rats sacrificed and imaged less than 4 weeks after surgery while the late group (n=19) was formed with the remaning rats (all sacrificed and studies at least 8 weeks after surgery). We relied on SPSS 15.0 to compare data. We used Paired T-test to compare the involved and uninvolved shoulders. Non parametric tests (Wilcoxon test and Mann–Whitney) were employed to compare between groups when necesary.

## Results

**Figure 1:**
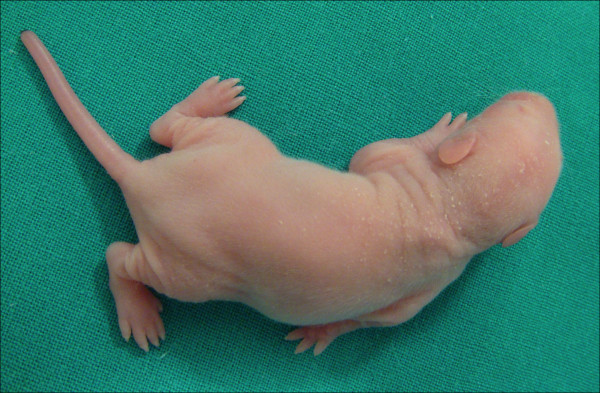
10-days-old rat (five days after surgery) showing shoulder paralysis and lack of elbow flexion after surgery.

**Figure 2:**
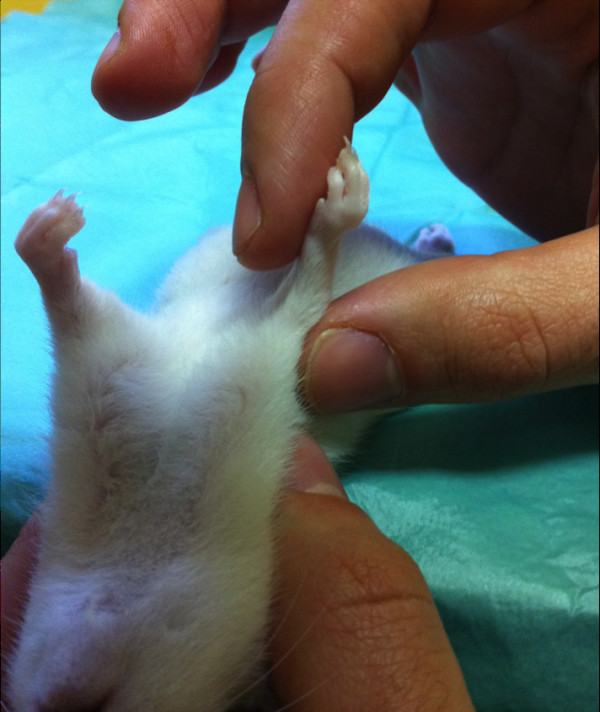
Axial vision of a rat showing a decrease of right passive shoulder external rotation 8 weeks after surgery.

The results are reproduced in Table 1. All animals showed right shoulder and elbow flexion paralysis after surgery, with an adduction and internal rotation posturing of the shoulder and lack of elbow flexion (Figure 1). There was a statistically significant decrease of passive external rotation in the involved shoulder compared to uninvolved shoulder (23.85°±27.5 vs 79.62°±3.8 respectively) (P<0,01) (Figure 2). When comparing early vs late group, no significant differences were found in mean passive shoulder external rotation (p=0.08).

**Table 1:** Shoulder rotation and MRI evaluation

	**Shoulder Passive external rotation (degrees)**	**Glenoid angle (degrees)**	**PHHA (%)**	**Humeral head sectional area (cm)**	**Subescapular thickness (cm)**	**Infraespinatus thickness (cm)**
Involved shoulder m(SD)	23.85	91.41	50.85	0.15	0.12	0.13(cm)
	(27.5)	(6.0)	(6.7)	(0.05)	(0.03)	(0.03)
Healthy shoulder	79.62	87.33	48.69	0.19	0.25	0.26
	(3.8)	(4.4)	(6.17)	(0.06)	(0.08)	(0.08)
p	0.000	0.000	0.058	0.000	0.000	0.000

**Figure 3:**
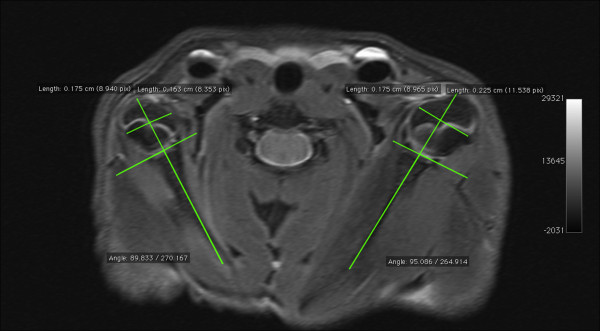
**Shoulder MRI 8 weeks after surgery showing symmetrical measures of glenohumeral joint alignment: glenoid version and PHHA.** This rat model does not reproduce the joint luxation found in children.

Mean glenoid version in the involved shoulder was 91.41°±6.0 and 87.33°±4.4 in the uninvolved. There was an statistically significant increase in the glenoid version in the involved shoulder, with an average increase of 4,08° (IC95% 2,31;5,83) (P<0,01). However, separate comparison between groups, showed a significative increase of glenoid anteversion in the early group, reaching 6,6° (IC95% 4.5° ; 8.6°) (P<0,01), but not in late group, 1,4° (IC95% -1.1°;3.9°) (P=0,4). Significant differences were found when comparing the increase of glenoid anteversion of both groups (P<0,01) (Figure 3). No statistically significant differences were found in PHHA between involved and healthy shoulder ( 50,85 ± 6.7% vs 48,69 ± 6.1% ) (P=0,1) (Figure 3).

**Figure 4:**
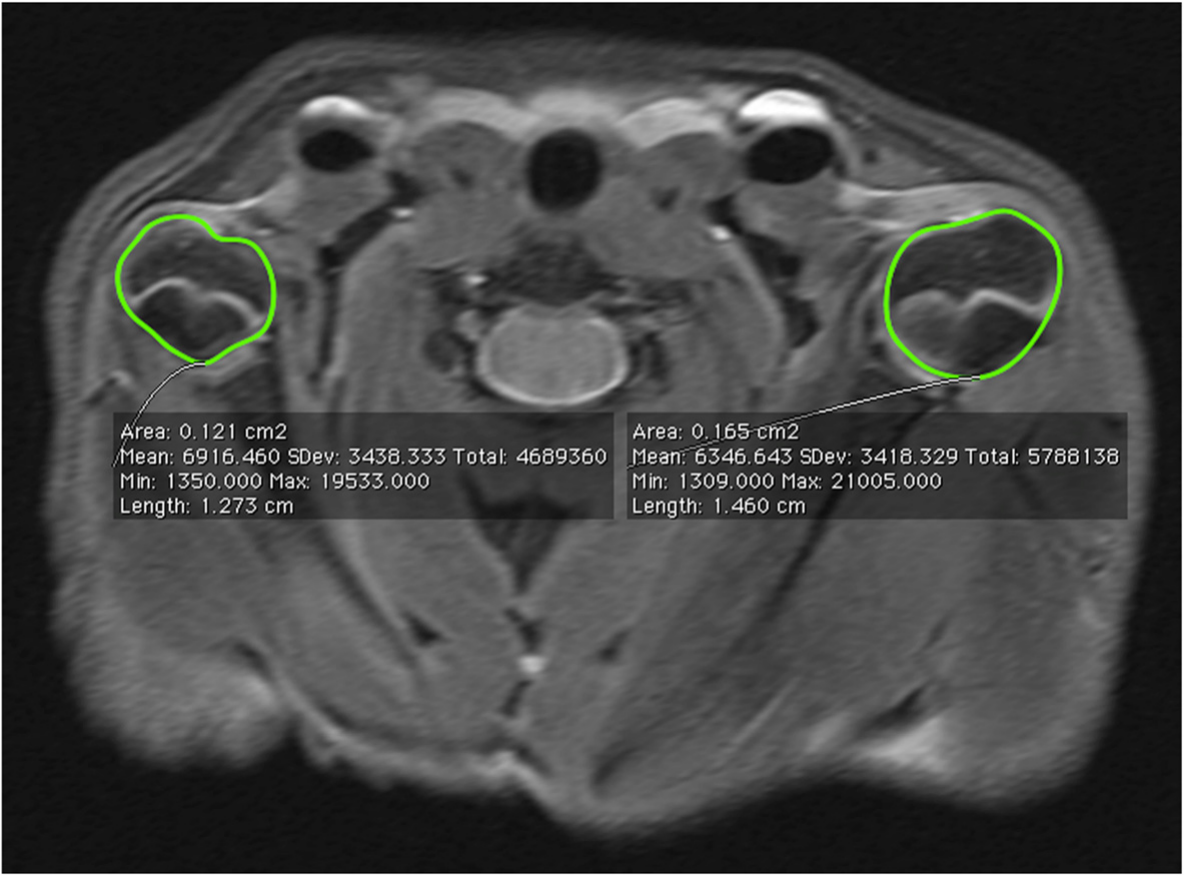
Shoulder MRI 8 weeks after surgery showing a decrease of humeral head sectional area that reproduces the joint hypoplasia found in children.

**Figure 5:**
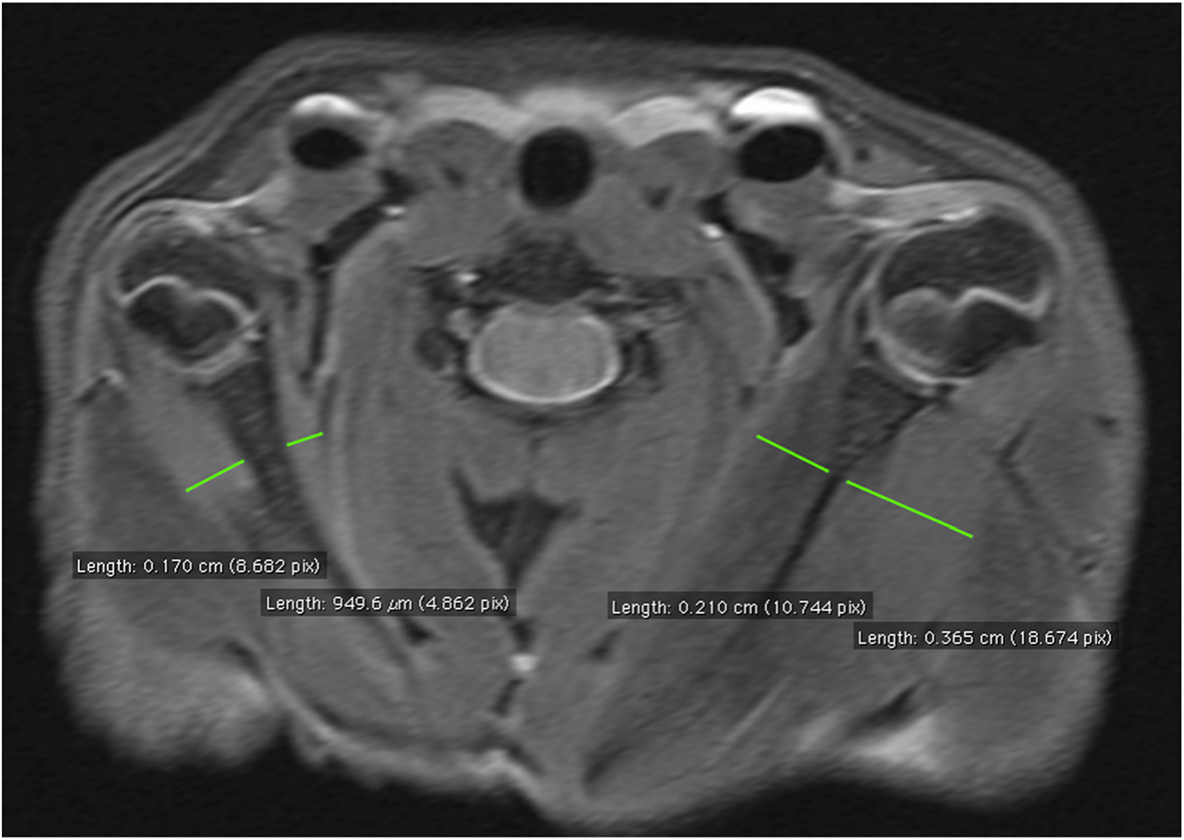
Shoulder MRI 8 weeks after surgery showing a marked atrophy of both infraspinatus and subscapular muscle that reproduces muscle changes found in children.

**Figure 6:**
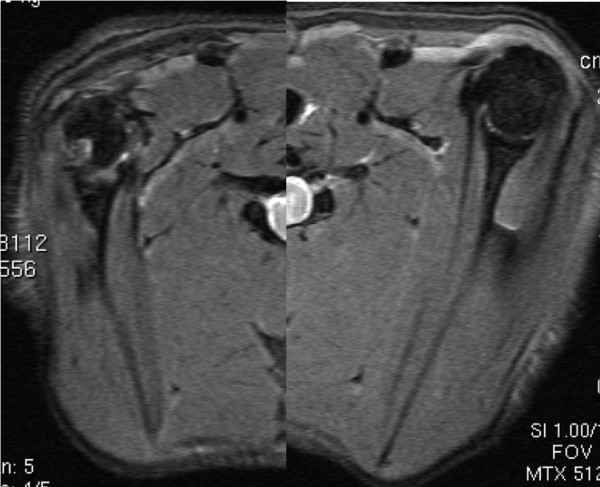
**Shoulder MRI 16 weeks after surgery showing a loss of relationship between the glenoid -which presents a normal version- and a marked deformed humeral head.** An advanced right glenohumeral joint hypoplasia is present. The distinctive shoulder dysplasia found in humans is absent.

Involved humeral head sectional area was significantly smaller than the healthy one with a mean size decrease of 19,9% (IC95% 16.9;23.3) (p<0.01). When comparing early and late groups, no significant differences were found in the decrease of humeral sectional area (p=0.73) (Figure 4). Involved infraspinatus muscle thickness was significatively smaller than the healthy side with a relative 46.3% (IC95 40.6;52) mean thickness decrease (P<0,01) (Figure 5). When comparing both groups, significantly more muscle thickness loss was found in the late group (56.9% (IC95% 50.6;63.3) vs. early group 36.21% (IC95 29.17;43.25) ( P<0,01)). Involved subscapular muscle thickness was significatively smaller than healthy one with a relative 52.5% (IC95 49.4;55.8) mean thickness decrease (P<0,01) (Figure 5). When comparing early and late groups, no significant differences were found in muscle thickness loss (p=0.02). When comparing loss of thickness between subscapular and infraspinatus muscle no statistical significant differences were found (P=0,02). However when comparing between early and late groups a statistically significative diference was found in the early group with a more severe atrophy in subscapular muscle (49.7% IC95% 44.6;54.7) than infraspinatus muscle (36.21% IC95% 29.17;43.25) p<0.01. Those differences were not found in the late group (p=0.55). Shoulder MRI of older rats showed severe deformity of the humeral head, the glenohumeral joint and the glenoid, but with no shoulder dislocation being found whatsoever (Figure 6).

## Discussion

The present study compares and contrasts our experimental findings to previous publications on shoulder structural changes after BPBP in animals and humans. Shoulder abnormalities are the most common long-term complication and the major cause of morbidity in BPBP. Nearly 30% of children with incomplete neuronal recovery go on into developing glenohumeral dysplasia [1,3,4,11-13]. An adequate preclinical model would be beneficial for the development of studies regarding pathogenesis, prevention or even treatment of this disabling disorder. The anatomy of the brachial plexus and its nerve distribution in rat is similar to that of humans and injury of upper brachial plexus roots leads to a clinical pattern similar to Erb´s palsy [14,15]. Although the murine model has been advocated as an adequate preclinical model of shoulder dysplasia following BPBP, our results lead us to disagree with this statement [8-10]. Shoulder anomalies following BPBP are characterized by muscle atrophy and contracture, as well as a progressive joint subluxation and joint hypoplasia [6]. Lack of shoulder external rotation and consequent joint deformities are progressive and appear early on. Initial changes can occur as early as the third month of life, while advanced deformities appear starting the second year [2,3]. Our model could show an early development of shoulder internal rotation contracture and joint changes. Natural history of shoulder joint and muscle changes after BPBP are been best followed with imaging studies [6,12,13]. MRI is the most accurate image tool to assess the pediatric glenohumeral joint due to its high percentage of cartilage. MRI would further allow a better standardization of radiologic measurements and muscle assessment [16,17]. This explains the choice of this imaging modality in our animal model. In fact, MRI was highly reliable in characterizing the normal anatomy and pathological shoulders changes in our rat model.

According to reported in MRI findings in children with BPBP, muscle atrophy is a common denominator in all rotator cuff muscles, but most prominently affects the subscapularis muscle [3,6,7]. The pathogenesis of the muscle changes that lead to the shoulder internal rotation contracture are barely understood with a few reports focusing on this topic [5-7,18]. The atrophy and contracture of the subscapularis muscle is thought to be the main contributor to shoulder contracture [19]. A recent experimental report concludes that these subscapularis changes are caused by muscle denervation-reinnervation. However recent clinical and radiological data supports that muscle imbalance around the shoulder might also play a role [19]. In our study, we chose to properly assess eventful muscle changes through the use of the MRI [8-10]. All our animals, similarly to the previously reported murine models, uniformly developed an early and severe shoulder internal rotation contracture [8-10]. Moreover, our model showed a severe atrophy of both subscapular and infraspinatus muscle. Infraspinatus muscle wasting seemed to appear more progressively than in the subscapular muscle, in which severe atrophy appeared very early. It must be emphasized that this phenomenon is similar to that encountered in children with shoulder dysplasia. In these, incomplete reinnervation leads to external rotator muscles atrophy and to an abnormal growth of the subscapularis muscle, further evolving into atrophy and contracture [20]. Further experimental studies including mechanical and histological assessment of periarticular muscles are recommended to define the exact pathogenesis of internal rotation contracture [5]. Though our model reproduced the muscular changes and the shoulder contracture, it failed to reproduce the typical glenoid deformity. Thus, the internal rotation contracture might not be the only causative factor for shoulder subluxation in BPBP. A possible explanation for these findings might be the quadripedal gait of the rat, with the shoulder being in a different mechanical disposition.

The glenoid version has been used to quantify the degree of glenoid deformity and the PHHA to quantify the degree of posterior humeral subluxation in children with shoulder dysplasia, as the typical human joint glenohumeral deformity combines a glenoid version change with humeral head subluxation [2,5]. The degree of change of both parameters is mutually proportional since the deformity occurs progressively and consequently. As a complete neurectomy of the upper trunk was performed (i.e. neurotmesis), a severe glenohumeral dysplasia combining a severe change in the glenoid version (or the development of a pseudoglenoid) and a severe humeral head subluxation, might be expected. However, our rats showed only a slight increase of glenoid anteversion, a finding that was simply present in the early group. Similarly, none of the previous murine models had reported severe glenoid version changes after a surgical neurotmesis of the upper brachial plexus trunk in the neonatal age [8-10]. Li’s first study speaks of a 10° increase of the glenoid anteversion while a contradictory 10° retroversion was obtained in his second study [8,9]. Kim’s mouse model also shows an 8.3° glenoid retroversion [10]. The discrepancy of the results between these studies might be due to the measurement method or to the fact that no intraobsever reliability test was conducted. In our study, the differences between early and late group might be due to these same reasons. We think that the shoulder dislocations reported in the mentioned papers do not correspond to a truly glenohumeral dysplasia but rather to an advanced degree of joint hypoplasia since they were associated to a very mild glenoid version change. The advanced joint hypoplasia might be related to the joint denervation that follows an upper trunk neurotmesis. Shoulder joint growth anomalies in children with incompletely recovered BPBP manifest by a delay in ossification nucleus growth and a decrease of humeral head size [2,4,21]. Abnormal mechanical transarticular loads due to muscle weakness and denervation of the physes and joint might play a role in joint underdevelopment. The marked joint hypoplasia that occurred in the oldest rats of our study, as well as those of the previously mentioned studies, might be explained by the severe joint denervation that follows the complete upper trunk neurectomy (i.e. neurotmesis). Both groups, (<4weeks and >8weeks), showed a significant decrease of the humeral head transverse sectional area but no statistical differences were found between them. However, the shoulder joint of the older rats (20 weeks) were very deformed. Severe deformity of the humeral head and loss of the articular space were present. These findings are similar to those reported in Li’s and Kim’s papers for the older animals (16 weeks old rats or 30 week old mice, respectively) [8-10]. All rats included in our study developed a marked shoulder contracture and subscapular changes in consonance with the changes occurring in children with BPBP. In addition, our model reproduced the joint hypoplastic changes found in human. However, our model failed to reproduce the typical human joint deformity with proportional glenoid retroversion and humeral head subluxation [1-4] as, in our opinion, did fail the previous experimental studies. A better understanding of the pathogenesis of shoulder internal rotation contracture after BPBP with a deep insight to the role of subscapular muscle changes are necessary to develop future strategies in the prevention and treatment of this disorder.

## Conclusions

The rat model might be an adequate preclinical model of shoulder contracture following BPBP but not of glenohumeral dysplasia. Studies regarding pathogenesis, prevention or treatment of shoulder contracture could be performed using this model.

## Competing interests

This work was funded by Instituto de Salud Carlos III, grant FIS PI10/01357 cofinanced by the European Regional Development Fund (ERDF), Fundacio ? Privada A. Bosch and by Fundação Santa-Maria-Silva. The authors declare that they have no conflict of interest.

## Authors' contributions

FS, BCD, KSH and HS conceived of the study, and participated in its design and coordination and draft the manuscript. MM, MME and FCG participated in the design of the study and performed the statistical analysis. IB and MVV carried out the radiological analysis. All authors read and approved the final manuscript.
